# An Enhanced Sherwood Number to Model the Hydrogen Transport in Membrane Steam Reformers

**DOI:** 10.3390/membranes11110805

**Published:** 2021-10-22

**Authors:** Maria Anna Murmura, Chiara Rocchetti, Maria Cristina Annesini

**Affiliations:** Dipartimento di Ingegneria Chimica Materiali Ambiente, Sapienza Università di Roma, Via Eudossiana 18, 00184 Roma, Italy; rocchetti.1690394@studenti.uniroma1.it (C.R.); mariacristina.annesini@uniroma1.it (M.C.A.)

**Keywords:** concentration gradients, 2D model, membrane reactors, hydrogen

## Abstract

It is well known that membrane reactors are inherently two-dimensional systems in which species concentrations vary as a consequence of both the reaction and permeation across the membrane, which occurs in the direction perpendicular to that of the main gas flow. Recently, an expression for an enhanced Sherwood number was developed to describe the hydrogen concentration gradients arising in methane steam-reforming membrane reactors as a consequence of the combined effect of hydrogen production, dispersion, and permeation. Here, the analysis is developed in further detail with the aim of (i) assessing the validity of the simplifying assumptions made when developing the 1D model and (ii) identifying the operating conditions under which it is possible to employ the 1D model with the enhanced Sherwood number.

## 1. Introduction

The use of membrane reactors has been attracting significant interest as a technology capable of allowing decentralized hydrogen production and coupling with solar heating technologies [1,2]. The description of such systems is made complicated by two fundamental aspects: the first is the interplay between the transport of mass and energy by convection and diffusion within the reactor, the effects of the reaction itself, and mass and energy transport through the hydrogen-permeable membrane; the second characteristic is that in these systems, hydrogen flows across the membrane in a direction perpendicular to that of the main gas flow, making the system inherently two-dimensional. In the past, many authors have resorted to 1D models to describe the behaviour of membrane reactors, effectively neglecting the presence of radial gradients [3,4,5]; however, it has been shown that such a solution often leads to results that are very different from those obtained in reality in terms of integral variables, such as the total amount of hydrogen permeating from the membrane per unit of time [6,7,8]. These observations have led to a tendency in recent years to resort to two- or three-dimensional models for membrane reactors [9,10] and only more rarely to one-dimensional models [11]. An extensive review of modelling approaches for packed bed membrane reactors, for the specific case of methane reforming may be found in [12].

To develop an accurate one-dimensional model without incurring in the difficulties of fully coupled two-dimensional models, it is therefore necessary to develop adequate correlations for the heat and mass transfer coefficients in order to account for the effect of resistances in the radial direction. In what follows, an isothermal reactor will be considered, and therefore, only the mass transfer coefficient will be discussed. It is well known that the mass transfer coefficient may be generally obtained from the Sherwood number, Sh, which is, in turn, correlated to the Reynolds and Schmidt numbers, describing, respectively, the momentum transport of the fluid and the ratio between mass and momentum diffusivity; however, when membrane reactors are involved, the mass transfer coefficient should also quantify the resistance to transport across the membrane and the effect of the reaction on concentration gradients. It is therefore evident that the need arises for correlations that differ from those commonly employed. To the best of our knowledge, at the moment, the attempt to obtain a novel expression for the enhanced Sherwood number has been made a few times for membrane separators [13,14], but only once, by some of the same authors of the present work, for membrane reactors [15].

The main objective of the present work has been to identify the operating conditions under which the prediction of the behaviour of membrane reactors for the steam reforming of methane through a 1D model with an enhanced Sherwood number equivalent to that obtained when employing a 2D model explicitly accounting for radial concentration gradients. The manuscript is divided as follows. In Section 2, the problem is detailed in its context. In Section 3, the modelling approach is described, and the main model equations are reported, including the expression of the enhanced Sherwood number. A discussion of the simplifying assumptions and their range of validity is reported in Section 4, while Section 5 presents the comparison between hydrogen recovery evaluated from the fully coupled 2D model and the 1D model making use of the enhanced Sherwood number.

## 2. Statement of the Problem

The problem has been studied through the following procedure:

1. An isothermal 2D model, in which mass and momentum transport in both the axial and radial directions were considered, described in detail in [16], was used as benchmark;
2. A simplified 2D model, derived in [17], was employed to derive an expression for the enhanced Sherwood number, valid when the performance of the system is not limited by hydrogen permeation across the membrane;
3. The range of operating conditions in which the expression of the Sherwood number is meaningful was identified;
4. A 1D model making use of the Sherwood number was employed to assess the performance of membrane reactors.

With regards to point (2), it is worth emphasizing that the operating conditions under which hydrogen transport across the membrane is not the limiting mass transport mechanism are the only ones for which it makes sense to employ a parameter that accounts for radial concentration gradients. When the membrane permeability is sufficiently low as to become the limiting factor, no radial concentration gradients are established within the packed bed, and a traditional 1D model is sufficient. It should be noted that this scenario is becoming of increasing interest as membranes characterized by significantly high hydrogen permeability are being developed [18].

The model was developed for a tube-in-tube reactor configuration, in which the total reforming reaction

(1) $${\mathrm{CH}}_{4}+2H_{2}O⇌{\mathrm{CO}}_{2}+4H_{2}$$

takes place. The catalyst is considered to be placed in the annular volume between the two concentric tubes. The outer wall is impermeable to all components, whereas a hydrogen-permeable membrane is placed on the outer wall of the innermost tube. In Pd-based membrane reactors, hydrogen selectivity is infinite, and permeation is described through Sieverts’ law

(2) $${\left.J_{h}\right|}_{membrane}=P_{m}\left(\sqrt{p_{H_{2}}^{r}}-\sqrt{p_{H_{2}}^{p}}\right)$$

where $P_{m}$ is the membrane permeability, and $p_{H_{2}}^{r}$ and $p_{H_{2}}^{p}$ are the partial pressures of hydrogen in the retentate and permeate side, respectively. The latter quantity has been set equal to zero, a condition that can be achieved in practice either by using high rates of sweep gas or by creating vacuum conditions in the permeate side. The radii of the inner and outer walls of the annular volume will be indicated by $R_{1}$ and $R_{2}$, respectively, from here on. The main equations of the full 2D model are reported in Table 1, along with the relevant boundary conditions, in both the dimensional and dimensionless forms.

The rate of methane consumption through the steam-reforming reaction may be described through the expression proposed by Wei and Iglesia [19]:

(3) $$r_{m}=kp_{m}\left(1-\eta \right)$$

where *k* is the reaction rate constant, $p_{m}$ is the partial pressure of methane, and the term $\left(1-\eta \right)$ quantifies the distance of the gas composition from the conditions of chemical equilibrium so that η takes on the form

(4) $$\eta =\frac{P}{K_{eq}}\frac{y_{h}^{4}y_{c}}{y_{m}y_{w}^{2}}$$

It is worth noting that the mass and momentum balance equations are fully coupled because velocity, as described by Darcy’s law for flow in porous media, is proportional to pressure gradients, and the dependence on gas composition and the total pressure of the mixture density have been considered.

The performance of these systems may be described through several parameters, such as the permeate flow rate and hydrogen recovery. The latter parameter, defined in Equation (5), is used here

(5) $$R_{h}=\frac{H{}_{2}\;\mathrm{permeate}\;\mathrm{flow}\;\mathrm{rate}}{H{}_{2}\;\mathrm{inlet}\;\mathrm{flow}\;\mathrm{rate}}$$

The maximum value of this parameter depends on the stoichiometry of the reaction considered. In the present case, $R_{h}$ would reach its maximum theoretical value if all the methane fed to the system were to be converted to hydrogen and the latter were to permeate completely

(6) $$R_{h,max}=1+4\frac{F_{CH_{4,in}}}{F_{H_{2,in}}}$$

The main characteristic groups that derive from the dimensionless formulation of the problem are the Peclet number

(7) $$Pe=\frac{Ul_{c}}{D}$$

which represents the ratio between the characteristic times of diffusion and convection; the Damkholer number

(8) $$Da=\frac{RTkl_{c}}{U}$$

which gives the ratio between the characteristic times of convection and reaction and the parameters γ and β that account for membrane permeability and pressure drops in the packed bed, respectively,

(9) $$\gamma =\frac{P_{m}RTP_{atm}^{-1/2}}{W_{h}}\frac{1}{U}$$

(10) $$\beta =\frac{\kappa }{\mu }\frac{P_{atm}}{l_{c}}\frac{1}{U}$$

The value of β is usually in the order of 10${}^{-3}$, which indicates that under the commonly adopted operating conditions, pressure drops are negligible. For this reason, the effect of β is not investigated in the remaining part of this work. The dimensionless pressure at the outlet will be referred to as $\alpha =P_{L}/P_{atm}$ from now on and will be taken as the reference operating pressure.

For the purposes of this work, it is worth noting that D is the generally non-isotropic dispersion tensor

(11) $$D=\left(\begin{array}{cc} D_{rr} & 0\\ 0 & D_{zz} \end{array}\right)$$

whose terms account for the interaction between diffusion and the small-scale convective flux related to the presence of the granular packing in the reactor. The effective Pe numbers in the axial and radial directions are defined, respectively, as

(12a) $$Pe_{eff,z}=\frac{l_{c}D_{z}}{U}$$

(12b) $$Pe_{eff,r}=\frac{l_{c}D_{r}}{U}$$

Based on the consideration reported in [20], the following relationship exists between the effective and molecular Pe numbers

(13a) $$\frac{1}{Pe_{eff,z}}=\frac{1}{\tau Pe}+\frac{\delta }{2}$$

(13b) $$\frac{1}{Pe_{eff,r}}=\frac{1}{\tau Pe}+\frac{\delta }{12}$$

where τ is the tortuosity factor, usually set to $\sqrt{2}$, and δ is the ratio between the characteristic dimension of the reactor, here chosen to be the difference between the outer and inner reactor radii, $l_{c}=R_{2}-R_{1}$, and the dimension of the packed bed particles.

The fully coupled 2D model described has been validated against experimental data available in the literature and has been found to be accurate over a wide range of operating conditions. The advantages of a complex modelling approach of this kind are those of providing interesting and useful insight on the mechanisms governing the performance of membrane reactors and of allowing the correct prediction of the behaviour of these systems, regardless of the values of the operating parameters. On the other hand, a high computational effort is required. In this context, it is useful to develop simplified models that nonetheless provide accurate information, at least in terms of integral quantities, such as the overall rate of hydrogen production. In a previous work [15], an expression for an enhanced Sherwood number, accounting not only for mass transfer by diffusion but also for the effect of the hydrogen-producing reaction and hydrogen permeation, was developed. The main assumptions made when developing such an expression were

1. Negligible axial dispersion;
2. Negligible radial convection;
3. Gas density independent of composition;
4. Excess steam in feed;
5. Local equilibrium conditions;
6. Infinite membrane permeability.

The assumptions that are most worthy of attention are those of infinite membrane permeability and negligible radial convection and will be discussed in detail in the following paragraphs.

## 3. Modelling Approach

The problem has been solved by considering that the ratio beetween the outer and inner reactor radii is sufficiently low as to allow the use of Cartesian coordinates. The radial coordinate has been rescaled as

(14) $$x=\frac{r-R_{1}}{R_{2}-R_{1}}$$

The first observation to be made is that, under the assumptions of uniform gas density, i.e., independent of gas composition, and of constant pressure, along with the observation that pressure drops in the reactor are negligible, the mass-average and molar-average velocities are the same, and mass balance equations may be written in terms of molar fractions. The mass balance equations for hydrogen and methane therefore read

(15a) $$\frac{\partial y_{h}}{\partial z}-\varepsilon \frac{\partial^{2}y_{h}}{\partial x^{2}}=\nu_{h}r_{m}$$

(15b) $$\frac{\partial y_{m}}{\partial z}-\varepsilon \frac{\partial^{2}y_{m}}{\partial x^{2}}=\nu_{m}r_{m}$$

where $\varepsilon =\frac{D_{rr}}{Pe}$, $r_{m}$ is the dimensionless rate of methane consumption, $\nu_{h}$ and $\nu_{m}$ are the stoichiometric coefficients of hydrogen and methane and are equal to 4 and −1, respectively. By defining the variable *Y* as the linear combination of the two molar fractions

(16) $$Y=\nu_{h}y_{m}-\nu_{m}y_{h}$$

the problem can be reduced to

(17) $$\frac{\partial Y}{\partial z}-\varepsilon \frac{\partial^{2}Y}{\partial x^{2}}=0$$

to be solved with the boundary conditions

(18a) $${\left.Y\right|}_{z=0}=Y^{0}=\nu_{h}y_{m}^{0}-\nu_{m}y_{h}^{0}$$

(18b) $${\left.\frac{\partial Y}{\partial x}\right|}_{x=1}=0$$

(18c) $${\left.Y\right|}_{x=0}=0$$

Equation (18c) is derived from the combination of four of the assumptions made, namely those of $p_{h}^{p}=0$, infinite membrane permeability, excess steam, and equilibrium conditions. The first two assumptions allow us to say that the partial pressure of hydrogen on the retentate side of the membrane must be zero. The latter two lead to the condition that any time the hydrogen concentration goes to zero, so should that of the limiting reactant, i.e., methane. The problem admits the following solution

(19) $$Y=2Y^{0}\sum_{l=0}^{\infty }\frac{1}{\lambda_{l}}exp\left(-\lambda_{l}^{2}\varepsilon z\right)sin\left(\lambda_{l}x\right)$$

where $\lambda_{l}=\frac{\pi }{2}\left(2l+1\right)$.

### 3.1. Sherwood Number

The Sherwood number is the dimensionless parameter commonly used to determine mass transport coefficients

(20) $$Sh=\frac{{\left.\frac{\partial y_{h}}{\partial r}\right|}_{membrane}}{\bar{y_{h}}-y_{h}^{M}}l_{c}=\frac{k_{c}l_{c}}{D_{er,H}}$$

where $D_{er,H}$ is the effective radial diffusion coefficient of hydrogen, $\bar{y_{H}}$ is the average value of the hydrogen molar fraction along the reactor’s cross-section, and $y_{H}^{M}$ is the value on the membrane. In the present case, the hydrogen concentration profile in the reactor depends on the combined effects of reaction, dispersion, convection, and permeation and therefore cannot be derived from common expressions proposed in the literature, which generally refer to situations in which there is neither reaction nor permeation.

It is possible to determine the expression of the Sherwood number from Equations (19) and (20), while keeping in mind that the problem is being studied with the assumption that $y_{H}^{M}=0$, as

(21) $$Sh\left(z\right)=-2Y^{0}\frac{\sum_{l=0}^{\infty }exp\left(-\lambda_{l}^{2}\varepsilon z\right)}{\bar{y_{h}}}\frac{1}{\nu_{m}}\frac{f_{in}}{W_{h}}$$

The need to evaluate the average hydrogen concentration on the reaction cross-section, $<y_{h}>$, to obtain the Sherwood number would make the use of the latter meaningless. It is, therefore, necessary to develop a simplified expression of the Sherwood number that is dependent only on the operating parameters. In [15], the following expression was proposed

(22) $$Sh\left(z\right)=Sh_{0}{\left(\frac{z}{z*}\right)}^{-0.45}$$

$Sh_{0}$ can be evaluated as

(23) $$Sh_{0}=-2Y^{0}\frac{\sum_{l=0}^{\infty }exp\left(-\lambda_{l}^{2}\varepsilon z^{*}\right)}{y_{h}^{0}}\frac{1}{\nu_{m}}\frac{f_{in}}{W_{h}}$$

where $z^{*}$ can be any value z>0, and the approximation has been made that $\bar{y_{h}\left(z^{*}\right)}=y_{h}^{0}$.

### 3.2. 1D Model

The expression for the Sherwood number reported in Equation (22) may be used in the following 1D model

(24a) $$\frac{d{\tilde{F}}_{h}}{d\tilde{z}}=4DaPy_{m}\left(1-\eta \right)-\frac{1}{\sigma -1}\frac{Sh\left(\tilde{z}\right)}{Pe_{eff,r}}y_{h}$$

(24b) $$\frac{d{\tilde{F}}_{m}}{d\tilde{z}}=-\;DaPy_{m}\left(1-\eta \right)$$

(24c) $$\frac{d{\tilde{F}}_{w}}{d\tilde{z}}=-2DaPy_{m}\left(1-\eta \right)$$

(24d) $$\frac{d{\tilde{F}}_{c}}{d\tilde{z}}\;\;=\;\;DaPy_{m}\left(1-\eta \right)$$

where $F_{i}$ is the molar flux of the *i*-th components, and the molar fractions appearing in Equation (24a‒d) are evaluated as

(25) $$y_{i}=\frac{F_{i}}{\sum_{j=1}^{n_{c}}F_{j}}$$

and σ is the ratio between the outer and inner radii of the reactor, i.e., $\sigma =R_{2}/R_{1}$.

## 4. Discussion of Simplifying Assumptions

### 4.1. Infinite Membrane Permeability

**Figure 1:**
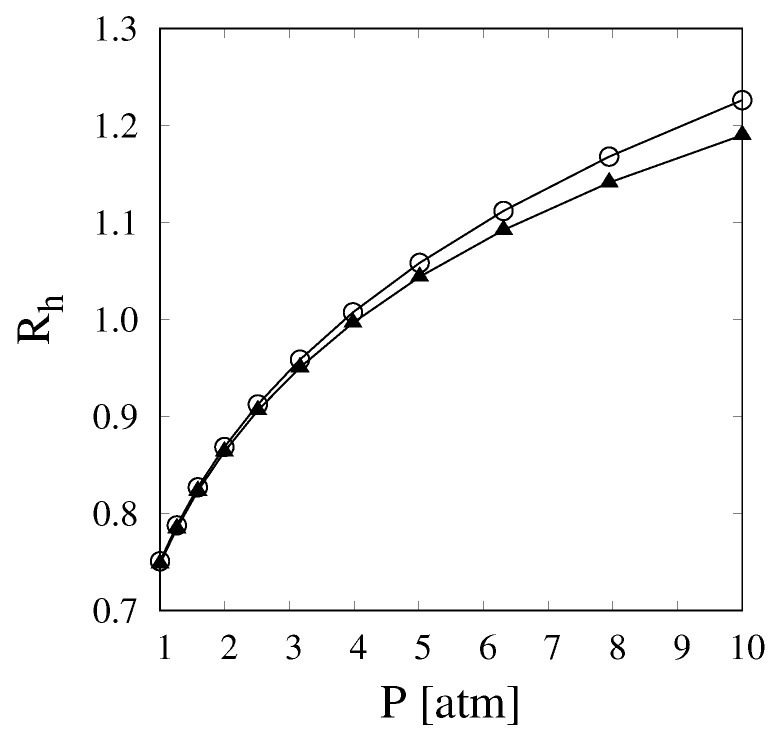
Hydrogen recovery, $R_{h}$, vs. pressure for a reactor operating with Da=1, Pe=100, and γ values of 1 (triangles) and 3 (circles).

In a previous work by some of the same authors [17], it was found that the behaviour of membrane reactors for the production of pure hydrogen can be divided into two main regimes: one in which the transport of hydrogen in the radial direction is limited by the membrane itself and one in which the resistance to mass transport within the packed bed is limiting. The existence of such regimes can be easily identified by examining the change in the slope of the curves of the hydrogen permeate flow rate vs. pressure under conditions of constant inlet velocity. The range of operating conditions for which it is possible to say that the behaviour of the system is limited by transport within the packed bed was identified in the previously cited work. When these conditions are met, it is possible to say that the membrane permeability is “infinite”, meaning that it offers no appreciable resistance and that the hydrogen concentration on the membrane wall always goes to zero. Generally speaking, the resistance to mass transport across the membrane becomes negligible in comparison to that of mass transport in the packed bed when the characteristic time of permeation is significantly lower than that of dispersion, i.e., when the product

(26) $$Pe_{eff,r}\times \gamma =\frac{P_{m}}{D_{r}}\frac{l_{c}RTP_{atm}^{1/2}}{W_{h}}$$

is significantly greater than unity. To precisely identify the range of operating conditions in which membrane permeability may be considered virtually “infinite”, the behaviour of the reactor was studied under a wide range of operating conditions in [17]. The space of operating parameters for which membrane resistance could be neglected is reported in Figure 1 of the same work and is summarized here in Table 2. Under realistic operating conditions, the product $Pe_{eff,r}\times \gamma$ takes on values between 20 and 50. It is clear that as the value of this parameter increases, the range of pressures in which the performance of the reactor is limited by transport within the packed bed becomes wider. Note that the Damkholer number is not reported because local equilibrium conditions are always assumed. In any case, for this latter assumption to be valid, the value of Da should always be greater than unity.

Figure 1 shows the hydrogen recovery, $R_{h}$, as a function of operating pressure. The curves have been evaluated for two different values of γ, namely 1 and 3, and for Da and Pe numbers equal to 1 and 100, respectively. With these sets of operating parameters, the product $Pe_{eff,r}\times \gamma$ is greater than 50; therefore, it should be possible to neglect the resistance offered by the membrane for pressure values lower than 11 atm. This assumption is supported by the fact that the two curves show little to no difference for pressures varying between 1 and 10 bar and that the difference is lowest for the lower values of pressure considered.

### 4.2. Effect of Radial Convection

If one were to consider a one-dimensional problem, the mass balance equations of hydrogen and methane would read

(27a) $$\nabla \cdot N_{h}=\nu_{h}r_{m}$$

(27b) $$\nabla \cdot N_{m}=\nu_{m}r_{m}$$

where $N_{h}$ and $N_{m}$ are the total fluxes of hydrogen and methane, respectively. Once again, it is possible to analyse the problem in terms of the linear combination of the molar fractions of hydrogen and methane

(28) $$Y=\nu_{h}y_{m}-\nu_{m}y_{h}$$

thus obtaining

(29) $$\nabla \cdot N_{Y}=0$$

For the generic *i*-th component, the total mass flux can be expanded as

(30) $$N_{i}=-D_{im}c_{TOT}\frac{dy_{i}}{dx}+y_{i}\sum_{j=1}^{n_{c}}N_{j}$$

where $c_{TOT}$ is the overall molar concentration of the mixture, which is assumed to be uniform, and $D_{im}$ is the dispersion coefficient of the *i*-th component in the mixture, which is considered to be the same for all the components present. The sum of the radial flux for all components, $\sum_{j}^{n_{c}}N_{j}$, is equal to the radial velocity and may be rewritten as

(31) $$\sum_{j=1}^{n_{c}}N_{j}=\left(1+r\right)N_{Y}$$

where *r* is the ratio between the sum of radial mass fluxes of all components and $N_{Y}$ and can be considered to be constant. Rewriting Equation (30) for the variable *Y*, taking into account Equation (31), yields

(32) $$N_{Y}\left[1-\left(1+r\right)Y\right]=-D_{Y}c_{TOT}\frac{dY}{dx}$$

which can be easily integrated between x=0 and x=δ, resulting in the following expression for the concentration profile

(33) $$Y=Y_{0}+\left(Y_{\delta }-Y_{0}\right)\frac{e^{\phi x/\delta }-1}{e^{\phi }-1}$$

where $Y_{0}$ and $Y_{\delta }$ are the values of *Y* in x=0 and x=δ, respectively, and

(34) $$\phi =\frac{N_{Y}\left(1+r\right)\delta }{D_{Y}c_{TOT}}$$

The diffusive flux in x=0 is given by

(35) $$-D_{Y}c_{TOT}{\left.\frac{dY}{dx}\right|}_{x=0}=-\frac{D_{Y}c_{TOT}}{\delta }\left(Y_{\delta }-Y_{0}\right)\frac{\phi }{e^{\phi }-1}$$

meaning that the Sherwood number, defined in Equation (20), is corrected by a factor

(36) $$f\left(\phi \right)=\frac{\phi }{e^{\phi }-1}$$

It is worth noting that

(37) $$\lim_{\phi \to 0}f\left(\phi \right)=1$$

which returns the expected result that when the radial velocity is low, the flux is purely diffusive.

The final expression of the Sherwood number should therefore be

(38) $$Sh\left(z\right)=Sh_{0}{\left(\frac{z}{z*}\right)}^{-0.45}\frac{\phi }{e^{\phi }-1}$$

where $Sh_{0}$ is unchanged with respect to the expression reported in Equation (23). From Equation (34), it is possible to see that ϕ is none other than a radial Peclet number, i.e., defined based on the radial velocity. The Peclet number used in the previous sections of this work is defined with respect to the axial velocity; therefore, it is possible to define ϕ as

(39) $$\phi =Pe\frac{v_{r}}{v_{z}}$$

**Figure 2:**
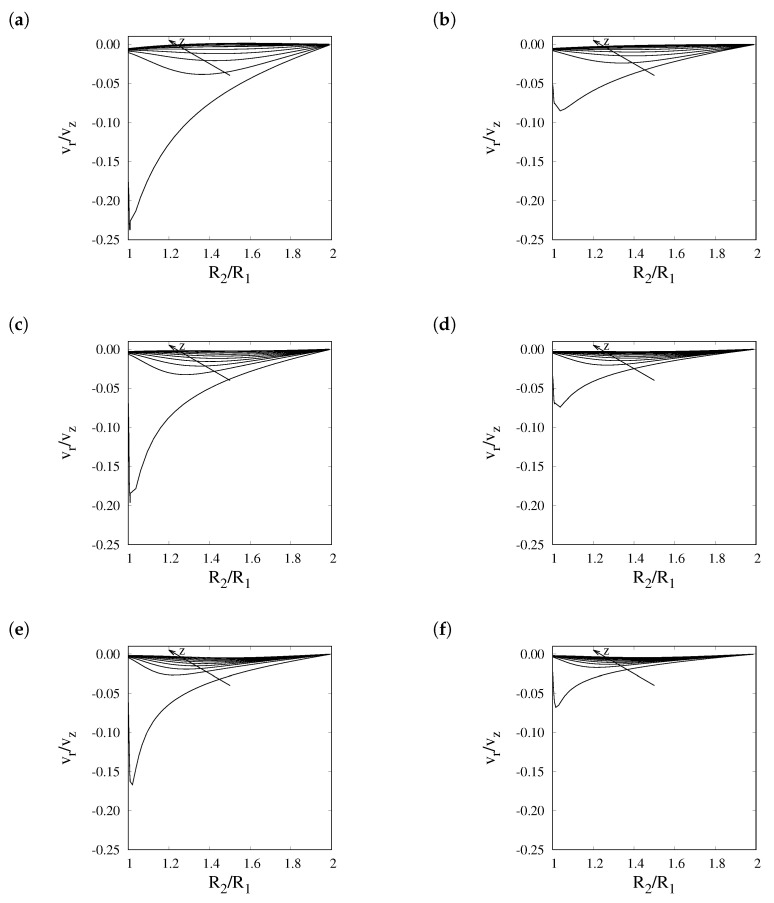
Ratio between the radial and axial components of velocity at 0.1, 10, 20, 30, 40, 50, 60, 70, 80, and 90% of the reactor length evaluated for γ = 5, *Da* = 1, and (**a**) *Pe* = 20, P = 1 atm (**b**) *Pe* = 20, P = 10 atm, (**c**) *Pe* = 50, P = 1 atm, (**d**) *Pe* = 50, P = 10 atm, (**e**) *Pe* = 100, P = 1 atm, and (**f**) *Pe* = 100, P = 10 atm.

Figure 2 shows the ratio between radial and axial components of velocity within the reactor under different operating conditions. It can be seen that the radial component of velocity is most significant in the region close to the reactor inlet, where it weighs about 10% of the axial velocity. Past the first 10% of the reactor length, the importance of radial convection drops significantly. Considering values of Pe ranging between 10 and 50, the maximum value of ϕ can vary between 0.1 and 0.5, and the correction factor varies between 0.95 and 0.78, suggesting that, under such range of operating conditions, radial convection may be neglected without committing significant errors in the evaluation of Sh and, consequently, of the flow rate of permeating hydrogen.

## 5. Results

**Figure 3:**
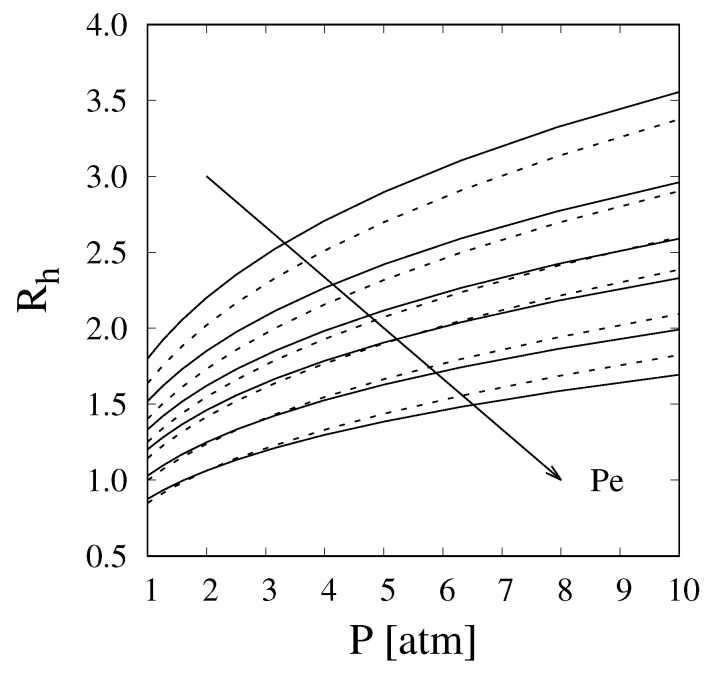
Hydrogen recovery vs. pressure evaluated through the full 2D model (solid lines) and 1D model with enhanced Sherwood number (dashed lines) at Da=5, γ=5, and Pe values of 20, 30, 40, 50, 70, and 100.

The 1D described in the previous paragraph was employed to evaluate the hydrogen recovery, defined in Equation (5), for values of Pe ranging from 20 to 100 and Da = 5. In Figure 3, these results were compared with those obtained from the fully coupled 2D under the same operating conditions and with γ = 5. It should be noted that lowering the value of γ to 1 does not lead to noticeable changes in the recovery predicted from the 2D model, as discussed in Section 4.1. For the 1D model, the flow rate of hydrogen permeating across the entire length of the reactor was evaluated as

(40) $$H_{2}\;\mathrm{permeate}\;\mathrm{flow}\;\mathrm{rate}=H_{2,in}-H_{2,out}+\underset{H_{2}\;\mathrm{produced}}{\underbrace{4\left(CH_{4,in}-CH_{4,out}\right)}}$$

It can be clearly seen that the recovery predicted from the simplified one-dimensional model is very close to that obtained from the two-dimensional model in which the different mass transport mechanisms were explicitly accounted for. As expected, the agreement is best for higher values of Pe and lower values of operating pressure, when dispersion significantly limits the transport of hydrogen in the radial direction. For instance, the deviation between the predicted recovery values is always lower than 5% at Pe=100. In any case, even for the worst-case scenario, i.e., low Pe and high pressures, the error in the recovery is l always lower than 10%. When considering the entity of this deviation, it is important to remember the significantly reduced computational cost of the 1D model with respect to its 2D counterpart and, most importantly, of the effort required to develop an accurate fully coupled 2D dimensional model.

**Figure 4:**
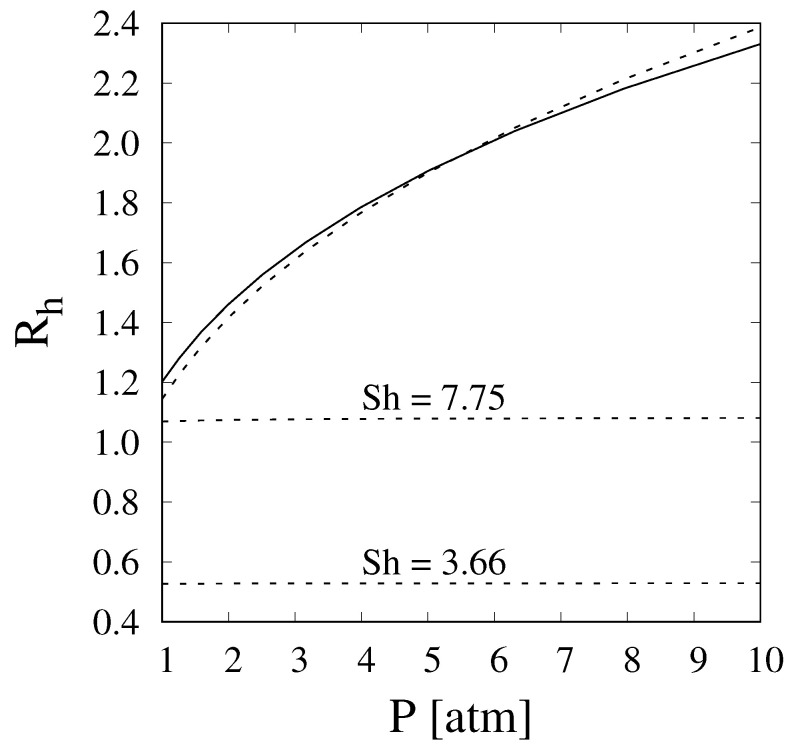
Comparison of hydrogen recovery vs. pressure evaluated through the full 2D model (solid line) and 1D model (dashed lines) with the enhanced Sherwood number (dashed lines) and the 1D model with fixed values of Sh of 3.66 and 7.75 at Da=5, γ=5, and Pe=50.

To appreciate the effect of evaluating the enhanced Sherwood number as conducted in the present work, i.e., accounting for the effect of a reaction and of a permeable membrane, Figure 4 shows a comparison of the recovery predicted with the full 2D model, the 1D model with the enhanced Sherwood number, and a 1D model in which the Sherwood number is set to fixed values of 3.66 and 7.75, as suggested in [21]. For the sake of clarity, the comparison has only been shown for a Pe value of 50, and all other conditions have been set to be equal to those of the results reported in Figure 3. It is clear that the enhanced Sherwood number obtained in the present work allows for a better prediction of hydrogen recovery in the steam-reforming reactor. In addition, it is evident that the effect of pressure needs to be taken into account explicitly.

## 6. Conclusions

Membrane reactors are inherently two-dimensional systems due to the transport of the permeating species in the direction perpendicular to that of the main gas flow. At the same time, the simultaneous presence of different transport mechanisms, namely convection, dispersion, reaction, and permeation, does not allow the use of traditional correlations for mass transfer coefficients. The description of membrane reactors, therefore, requires the use of 2D models or 1D models making use of mass transfer coefficients evaluated specifically for these systems. The present work presents the development of one such correlation, along with a discussion of the simplifying assumptions made, and of the operating conditions under which they are valid. The work was developed for the case of methane steam reforming but may be extended to other reacting systems. It was shown that the behaviour predicted by the 1D model with the proposed expression for the Sherwood number follows that of the 2D model closely, in which the different transport mechanisms are explicitly accounted for. Under the same operating conditions, the use of Sherwood numbers proposed for packed bed reactors leads to significant deviations between the results predicted with 2D and 1D models. A useful tool is therefore developed to assess the performance of membrane reactors with a high precision and low computational cost. Future research should be directed in the development of a model that also accounts for the thermal effects of the reaction and reactor heating.

## Figures and Tables

**Table 1:** Main equations and boundary conditions.

Dimensional Formulation		
**Continuity, Momentum, and Mass**		
	$\nabla \cdot \left(\rho v\right)=0$	
	$v=-\frac{\kappa }{\mu }\nabla P$	
	$\nabla \cdot \left(-\frac{\kappa }{\mu }fP\;\omega_{i}\nabla P-fP\;D\cdot \nabla \omega_{i}\right)=r_{i}$	
**Boundary conditions**		
z=0	$v_{z}=U$	$\omega_{i}=\omega_{i}^{0}$
z=L	$P=P_{L}$	$\frac{\partial \omega_{i}}{\partial z}=0$
$r=R_{1}$	$\frac{1}{RT}\left(-\frac{\kappa }{\mu }\nabla P\right)\cdot n_{1}=P_{m}\left(\sqrt{\frac{\omega_{h}}{W_{h}fP}}\right)$	
	$\frac{1}{RT}\left(-\frac{\kappa }{\mu }\omega_{h}\nabla P-D\cdot \nabla \omega_{h}\right)\cdot n_{1}=P_{m}\left(\sqrt{\frac{\omega_{h}}{W_{h}fP}}\right)$	
	$\frac{1}{RT}\left(-\frac{\kappa }{\mu }\omega_{i}\nabla P-D\cdot \nabla \omega_{i}\right)\cdot n_{1}=0\;,\;i\neq h$	
$r=R_{2}$	$\nabla P\cdot n_{2}=0$	
	$\frac{1}{RT}\left(-\frac{\kappa }{\mu }\omega_{i}\nabla P-D\cdot \nabla \omega_{i}\right)\cdot n_{2}=0$	
**Dimensionless Formulation**		
**Continuity, Momentum, and Mass**		
	$\tilde{\nabla }\cdot \left(\tilde{f}\tilde{P}\tilde{\nabla }\tilde{P}\right)=0$	
	$\tilde{v}=-U_{c}\tilde{\nabla }\tilde{P}$	
	$\tilde{\nabla }\left(-\frac{1}{\beta }\tilde{f}\tilde{P}\omega_{h}\tilde{\nabla }\tilde{P}-\frac{1}{Pe}\tilde{f}\tilde{P}\tilde{D}\tilde{\nabla }\omega_{h}\right)={\tilde{r}}_{i}$	
**Boundary Conditions**		
$\tilde{z}=0$	${\tilde{v}}_{z}=1$	$\omega_{i}=\omega_{i}^{0}$
$\tilde{z}=L/R_{1}$	$\tilde{P}=P_{L}/P_{atm}$	$\frac{\partial \omega_{h}}{\partial \tilde{z}}=0$
$\tilde{r}=1$	$\frac{1}{\beta }\tilde{\nabla }\tilde{P}\cdot n_{1}=-\gamma \left(\sqrt{\frac{\omega_{h}}{\tilde{f}\tilde{P}}}\right)$	
	$\left(\frac{1}{\beta }\omega_{h}\tilde{\nabla }\tilde{P}+\frac{1}{Pe}\tilde{D}\cdot \tilde{\nabla }\omega_{h}\right)\cdot n_{1}=-\gamma \left(\sqrt{\frac{\omega_{h}}{\tilde{f}\tilde{P}}}\right)$	
	$\left(\frac{1}{\beta }\omega_{i}\tilde{\nabla }\tilde{P}+\frac{1}{Pe}\tilde{D}\cdot \tilde{\nabla }\omega_{i}\right)\cdot n_{1}=0\;\;i\neq h$	
$\tilde{r}=R_{2}/R_{1}$	$\tilde{\nabla }\tilde{P}\cdot n_{2}=0$	
	$\left(\frac{1}{\beta }\omega_{i}\tilde{\nabla }\tilde{P}+\frac{1}{Pe}\tilde{D}\cdot \tilde{\nabla }\omega_{i}\right)\cdot n_{2}=0$	

$n_{1}$ and $n_{2}$ are local unit vectors normal to the surface and oriented outward the reaction volume.

**Table 2:** Range of operating conditions in which the membrane reactor operates in a transport-controlled regime.

${\mathrm{Pe}}_{\mathrm{eff},r}\times \gamma$	P (atm)
6	<1.2
13	<3.5
26	<10
50	<11

## Abbreviations

$a_{M}^{l}$	membrane area per unit length of reactor (m)
D	diffusion coefficient (m${}^{2}$/s)
D	effective dispersion tensor (m${}^{2}$/s)
*f*	average molar weight (kg/mol)
$I_{h}$	inlet flow rate of hydrogen (kg${}_{H_{2}}$/s)
$J_{h}$	hydrogen mass flux (kg${}_{H_{2}}/($m${}^{2}\cdot$ s))
*k*	rate constant of the methane reforming reaction (mol/(m${}^{3}\;\cdot \;$s·Pa))
$K_{eq}$	equilibrium constant of the methane reforming reaction (Pa${}^{2}$)
*L*	reactor length (m)
n	unit vector normal to the membrane surface $\left(-\right)$
$N_{i}$	total molar flux of the *i*-the component (mol/m${}^{2}$s)
*P*	pressure (Pa)
$P_{i}$	partial pressure of the *i*-th component (Pa)
$P_{L}$	outlet pressure (Pa)
$P_{m}$	membrane permeability (kg${}_{H_{2}}/$(m${}^{2}\;\cdot \;$s· Pa${}^{0.5}$))
*r*	radial coordinate (m)
$r_{i}$	volume-specific mass rate of production of the *i*-th component (kg/(m${}^{3}\cdot$ s · Pa))
R	gas constant (J/(mol · K))
$R_{h}$	hdyrogen recovery
$R_{1}$	inner reactor radius (m)
$R_{2}$	outer reactor radius (m)
$r_{m}$	volume-specific molar rate of methane consumption (mol/(m${}^{3}\;\cdot \;$s· Pa))
*S*	reactor cross-section (m${}^{2})$
Sh	Sherwood number $\left[-\right)$
*T*	temperature (K)
*U*	inlet gas velocity (m/s)
v	mass average velocity (m/s)
$W_{i}$	molar weight of the *i*-th component (kg/mol)
*x*	re-scaled radial coordinate $x=\left(r-R_{1}\right)/\left(R_{2}-R_{2}\right)$
*y*	molar fraction $\left(-\right)$
*Y*	linear combination of hydrogen and methane molar fractions $\left(-\right)$
*z*	axial coordinate
**Greek Symbols**	
β	ratio between characteristic and inlet velocities$\left(\kappa P_{atm}/\left(\mu R_{1}U\right)\right)\;\left(-\right)$
ε	$\frac{{\tilde{D}}_{rr}}{Pe}$
η	proximity to reaction equilibrium $\left(-\right)$
κ	packed bed permeability (m${}^{2})$
μ	gas viscosity (Pa · s)
ρ	gas density (kg/m${}^{3}$)
$\rho_{i}$	density of the *i*-th component (kg/m${}^{3}$)
σ	geometric ratio, $R_{2}/R_{1}$
$\nu_{i}$	stoichiometric coefficient of the *i*-th component $\left(-\right)$
$\omega_{i}$	mass fraction of the *i*-th component $\left(-\right)$
Ω	linear combination of hydrogen and methane mass fractions $\left(-\right)$
**Dimensionless Parameters**	
Da	Damkholer number $\left(RTkR_{1}/U\right)$
${\tilde{D}}_{rr}$	dimensionless radial dispersion $\left(D_{rr}/D\right)$
$\tilde{D}$	dimensionless dispersion $\left(D/D\right)$
Pe	Peclet number $\left(UR_{1}/D\right)$
α	dimensionless outlet pressure $\left(P_{L}/P_{U}\right)$
γ	dimensionless permeability parameter $\left(P_{m}RTP_{atm}^{-1/2}/W_{h}U\right)$
**Subscripts and Superscripts**	
*c*	carbon dioxide
*h*	hydrogen
*m*	methane
*w*	water
*M*	membrane
